# Accelerating ab initio melting property calculations with machine learning: application to the high entropy alloy TaVCrW

**DOI:** 10.1038/s41524-024-01464-7

**Published:** 2024-11-29

**Authors:** Li-Fang Zhu, Fritz Körmann, Qing Chen, Malin Selleby, Jörg Neugebauer, Blazej Grabowski

**Affiliations:** 1 Department for Computational Materials Design, Max Planck Institute for Sustainable Materials, Max-Planck-str.1, 40237 Düsseldorf, Germany; 2 Institute for Materials Science, University of Stuttgart, Pfaffenwaldring 55, 70569 Stuttgart, Germany; 3 Interdisciplinary Centre for Advanced Materials Simulation (ICAMS), Ruhr-Universität Bochum, 44801 Bochum, Germany; 4 Thermo-Calc Software AB, Råsundavägen 18, SE-169 67 Solna, Sweden; 5 Department of Materials Science and Engineering, KTH Royal Institute of Technology, SE-100 44, Stockholm, Sweden

**Keywords:** Computational methods, Metals and alloys, Phase transitions and critical phenomena

## Abstract

Melting properties are critical for designing novel materials, especially for discovering high-performance, high-melting refractory materials. Experimental measurements of these properties are extremely challenging due to their high melting temperatures. Complementary theoretical predictions are, therefore, indispensable. One of the most accurate approaches for this purpose is the ab initio free-energy approach based on density functional theory (DFT). However, it generally involves expensive thermodynamic integration using ab initio molecular dynamic simulations. The high computational cost makes high-throughput calculations infeasible. Here, we propose a highly efficient DFT-based method aided by a specially designed machine learning potential. As the machine learning potential can closely reproduce the ab initio phase-space distribution, even for multi-component alloys, the costly thermodynamic integration can be fully substituted with more efficient free energy perturbation calculations. The method achieves overall savings of computational resources by 80% compared to current alternatives. We apply the method to the high-entropy alloy TaVCrW and calculate its melting properties, including the melting temperature, entropy and enthalpy of fusion, and volume change at the melting point. Additionally, the heat capacities of solid and liquid TaVCrW are calculated. The results agree reasonably with the CALPHAD extrapolated values.

## Introduction

Discovering novel high entropy alloys (HEAs) with exceptional performance has ushered in a new era for materials design^1–3^. The melting temperature is a crucial parameter in the search for such materials. For instance, a correlation between a high melting point and elevated temperature strength has been identified in refractory complex concentrated alloys^4^. Besides the melting temperature, other melting properties, such as enthalpy and entropy of fusion, and volume change at the melting point, are also crucial for constructing phase diagrams and developing novel materials. However, experimental measurements on these properties, even for unary refractory materials, face severe challenges due to high melting points, often resulting in very scattered experimental data, if available at all. Additionally, the vast compositional space of HEAs makes systematic experimental screening of promising candidates impractical.

Several computational methods for melting point predictions have been developed using empirical potentials, machine learning potentials, and density functional theory (DFT)^5–7^. Calculations on other melting properties, such as entropy and enthalpy of fusion, volume expansion from solid to liquid at the melting point, and thermodynamic properties of the liquid phase (especially the liquid heat capacity), are, however, limited. They require access to the free energy surface of both solid and liquid, including all relevant physical contributions, such as vibrational entropy, including the anharmonic contribution, and electronic entropy, including the electron-vibration coupling. These physical contributions significantly impact the thermodynamic properties of both the solid and liquid phases, thereby affecting the predicted melting properties^8–10^.

One approach, capable of including these contributions and considered a gold standard for such calculations due to its high accuracy, is the ab initio free energy approach within the DFT framework^11–17^. In this approach, Gibbs energies of the solid and liquid phases are explicitly calculated, and the crossing point of the solid and liquid Gibbs energies determines the melting point. The solid and liquid thermodynamic properties can also be extracted from the Gibbs energies. However, achieving a high accuracy entails a significant computational cost, primarily due to the typically involved expensive ab initio molecular dynamics (MD) simulations.

Methods for speeding up ab initio solid free energy calculations have been under active development. A recent advancement in this direction is based on dedicated machine learning potentials to entirely avoid the costly ab initio MD calculations^18,19^. The ab initio accuracy is then achieved by performing static DFT calculations on a few snapshots generated by the machine learning potential using free energy perturbation theory^20^. This approach is called “direct upsampling" and has shown significantly improved computational efficiency compared to the previous ab initio MD-based approaches using thermodynamic integration^3,21,22^.

For ab initio liquid free energy calculations, due to the randomly distributed positions of the atoms, a static lattice reference is missing. Therefore, the liquid free energy calculation relies on designing a good reference to closely reproduce the ab initio liquid phase space and on performing thermodynamic integration calculations based on ab initio MD. A recent development for speeding up the liquid free energy calculations is the TOR-TILD (*two-optimized reference thermodynamic integration using Langevin dynamics*) methodology^16^. It has demonstrated remarkable efficiency for calculating elemental materials and binary alloys^10,16,17^ by employing two specially designed EAM potentials as references. However, when going to multi-component alloys, its computational speed significantly slows down. The reason is that the large compositional space of multi-component alloys results in many different atomic structures. The requirements to fit an efficient reference to reach the same accuracy as for unary systems are thus more critical. In this case, EAM potentials lose their power to accurately describe the ab initio liquid system.

Here, we propose an efficient ab initio based approach for liquid free energy calculations inspired by direct upsampling for solid phases. A key ingredient to the proposed approach is a specially designed machine-learning interatomic potential. This potential can be any machine learning model as long as it shows excellent performance regarding accuracy and efficiency, e.g., the recently developed atomic cluster expansion (ACE) potentials^23^ or moment tensor potentials (MTPs)^24,25^. In the present work, MTP is used. As the phase-space distribution of the specially designed MTP closely overlaps with the ab initio distribution for the liquid, even for multi-component materials, it becomes possible to entirely skip the expensive ab initio MD simulations and directly up-sample to the DFT level using a few snapshots from the MTP generated trajectories based on free energy perturbation.

We use the TaVCrW HEA as a prototype system to demonstrate the performance of the proposed methodology. We compare the respective results with those obtained from the original TOR-TILD method using two EAM potentials and a hybrid TOR-TILD approach with one EAM potential and one MTP. The free energy and thermodynamic properties of solid TaVCrW are computed using the direct upsampling approach. Combining the thermodynamic properties of solid and liquid TaVCrW, the melting properties of TaVCrW are obtained and compared to available CALPHAD data, including the heat capacity and bulk modulus of the liquid phase. GGA-PBE and LDA exchange-correlation functionals are used, and their performance in predicting melting properties for the TaVCrW alloy is discussed.

## Results

### General overview of the methodology

A key ingredient to the proposed methodology is an optimized machine-learning potential (here, an MTP) that replaces one of the two classical EAM potentials utilized within the TOR-TILD method^16^. This replacement allows us to remove the computationally very expensive DFT-based thermodynamic integration and to replace it with efficient free-energy perturbation theory. This is possible because of the high accuracy of the MTP, which provides a similar phase-space distribution as DFT and thus facilitates a quick convergence within free-energy perturbation calculations. It is important that only one of the classical potentials is replaced by the MTP. The other classical EAM potential is needed in order to guarantee efficient coexistence calculations, as MD calculations using such classical potentials are generally 30–50 times faster than those using machine learning potentials. In particular, coexistence calculations require large supercells, long sampling times, and many statistically independent runs in order to guarantee a precise melting point prediction. However, they do not need to be accurate (i.e., close to DFT) because, in the later steps of the proposed method, accuracy is restored by thermodynamic integration to the MTP and perturbation theory to DFT. Overall, we achieve an optimized hierarchically structured approach that profits from both the accuracy of machine learning potentials and the speed of classical potentials.

**Fig. 1:**
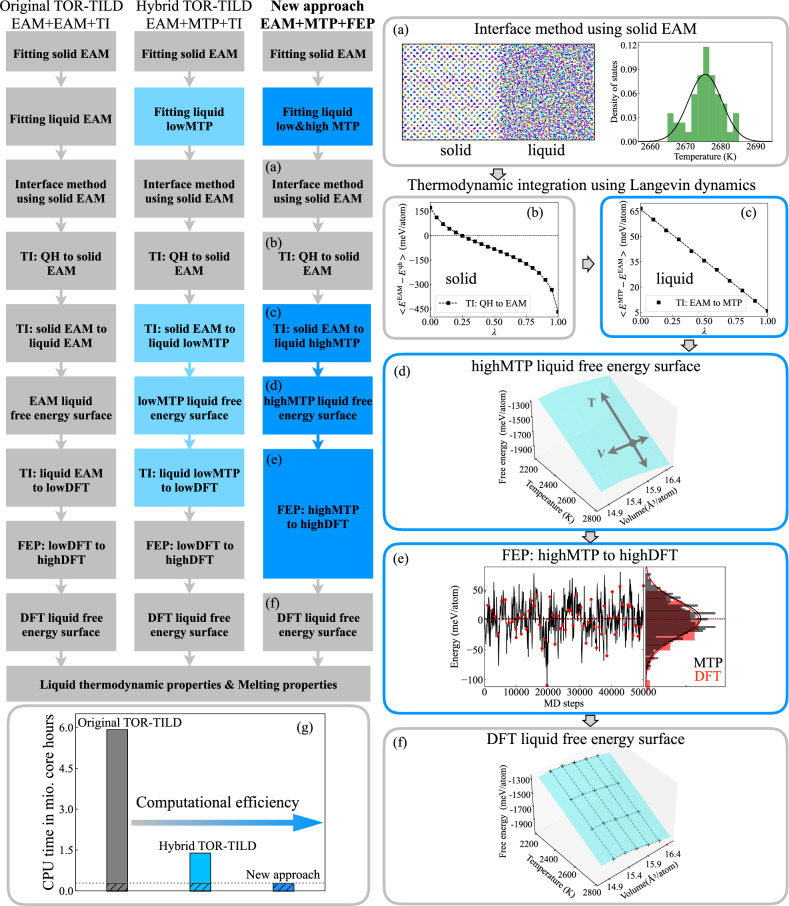
Schematic of the proposed method for liquid free energy calculations in comparison to TOR-TILD. Steps modified with respect to the original and hybrid TOR-TILD method are marked in light and dark blue, respectively. The workflow for the new approach is emphasized in (**a**)–(**f**). **a** Shows the solid-liquid interface structure used for predicting the melting point of the EAM and the resulting Gaussian distribution of the melting points. **b**, **c** Depict thermodynamic integration from an effective quasiharmonic (QH) reference to EAM and from EAM to MTP at the predicted melting point. **d** Represents the MTP liquid free energy surface calculated via integration of the internal energy and pressure along the temperature and volume dimensions starting from the free energy value (gray dot) obtained in (**c**). **e** Displays the MD trajectory of MTP (black line) and DFT snapshot energies (red dots) with their Gaussian distributions and mean values (dashed lines). **f** Is the DFT liquid free energy surface with the DFT data points marked by black crosses. See “Methods” section for additional details. **g** Compares the computational efficiency of the three methods.

The proposed approach is contrasted with the original TOR-TILD method and with a hybrid TOR-TILD method in Fig. 1. The original TOR-TILD method is additionally labeled as EAM+EAM+TI, indicating the usage of two classical EAM potentials (one for the coexistence calculations and one for the optimized description of the liquid) and thermodynamic integration (TI) to reach DFT accuracy. The hybrid TOR-TILD method is also labeled by EAM+MTP+TI to indicate the replacement of the liquid EAM by the MTP, while the thermodynamic-integration step is preserved. We introduce this hybrid TOR-TILD method here to facilitate a systematic comparison of the methods and a systematic investigation of the achievable time savings (Section “Efficiency and accuracy”). Finally, the new method is labeled by EAM+MTP+FEP, clarifying the additional replacement of the expensive thermodynamic integration by free energy perturbation (FEP) theory. Following the respective columns in Fig. 1, one can identify the steps that are modified with respect to the original TOR-TILD method (gray → light blue → blue). For the newly proposed method, the workflow is further emphasized by the subfigures (a) to (f).

The key equation of the new approach expresses the target free energy of the liquid *F*^liquid^ as:

1 $$\begin{array}{lll}{F}^{{\rm{liquid}}}(V,T)=\overbrace{{F}_{{\rm{EAM}}}^{{\rm{liquid}}}+{{\Delta }}{F}_{{\rm{EAM\to }}\rm{MTP}}^{{\rm{liquid}}}}^{{\text{at}}\left({V}_{{\rm{EAM}}}^{{\rm{m,liquid}}},{T}_{{\rm{EAM}}}^{{\rm{m}}}\right)}\\\qquad\qquad\qquad+\underbrace{{{\Delta }}{F}_{{\rm{MTP}}}^{{\rm{liquid}}}(V,T)+{{\Delta }}{F}_{{\rm{MTP\to }}{\rm{DFT}}}^{{\rm{liquid}}}(V,T)}_{{\mathrm{on}}\, {\mathrm{a}}\,(V,T)\,\text{mesh}}.\end{array}$$

The first term ${F}_{{\rm{EAM}}}^{{\rm{liquid}}}$ describes the liquid free energy of the classical EAM potential, the one that is retained in the proposed method and that works as the initial reference. The computation of this free-energy term is the same as in the original TOR-TILD method via the interface (aka coexistence) method (Fig. 1a), thermodynamic integration for the solid from a quasiharmonic reference to the EAM (Fig. 1b and Eq. (2)), and the equality of the solid and liquid Gibbs energies at the melting point. The second term ${\Delta F}_{{\rm{EAM}}\to{\rm{MTP}}}^{{\rm{liquid}}}$ is the difference between the EAM potential and MTP. In the original TOR-TILD method, the second potential is a classical EAM potential, but is replaced here by a machine learning potential, i.e., MTP. The difference ${\Delta F}_{{\rm{EAM}}\to{\rm{MTP}}}^{{\rm{liquid}}}$ can be very efficiently obtained by thermodynamic integration (Fig. 1c and Eq. (3)), which has fewer requirements with respect to system size and time sampling than the interface method. Both of the terms ${F}_{{\rm{EAM}}}^{{\rm{liquid}}}$ and ${{\Delta }}{F}_{{\rm{EAM\to }}\rm{MTP}}^{{\rm{liquid}}}$ are calculated at the melting temperature and corresponding volume of the EAM potential, ${T}_{{\rm{EAM}}}^{{\rm{m}}}$ and ${V}_{{\rm{EAM}}}^{{\rm{m}},{\rm{liquid}}}$. The next term in Eq. (1), ${{\Delta}}{F}_{{\rm{MTP}}}^{{\rm{liquid}}}(V,T)$, unfolds the liquid free energy surface in the volume and temperature dimensions. For that purpose, integration of the internal energy and pressure along the temperature and volume axis is performed (Fig. 1d and Eqs. (4), (5)). As for thermodynamic integration, also this calculation step has fewer requirements with respect to system size and time sampling than the interface method. The last term ${{\Delta}}{F}_{{\rm{MTP\to }}{\rm{DFT}}}^{{\rm{liquid}}}$ corrects the free energy obtained by the MTP potential to DFT accuracy. In the original TOR-TILD method, a thermodynamic integration is required in this last step because, in general, the accuracy of classical potentials is not enough to guarantee a converged perturbative approach. In the here proposed method, we can instead rely on free energy perturbation (Fig. 1e and Eq. (6)) for this term because of the sufficiently high accuracy of the MTP. The utilized MTP is a conventional machine-learning potential, i.e., one that captures the vibrational degrees of freedom but not the electronic ones. Future extensions with the recent electronic MTPs^26^ are possible but beyond our scope. The electronic contribution and the important impact of vibrations on it are, nevertheless, fully included in the present method by employing finite temperature DFT within the perturbation calculations. The final free energy surface (Fig. 1f) allows us to extract DFT accurate melting properties.

It is important to stress that the MTP involved in the proposed method is solely optimized for an accurate description of the part of the DFT phase space representative of the liquid. It is not guaranteed that this MTP is transferable to other parts of the DFT phase space. The MTP functions only as a very efficient bridge to obtain DFT-accurate thermodynamics of the liquid phase.

### Efficiency and accuracy

**Fig. 2:**
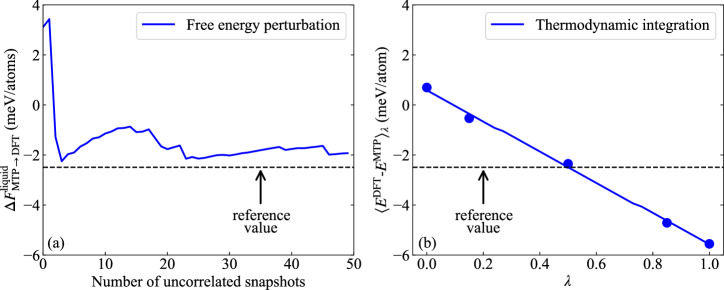
Free energy difference between MTP and DFT using GGA-PBE for liquid TaVCrW at *T* = 2400 K and *V* = 16.24 Å^3^/atom. **a** Calculated from free energy perturbation. **b** Calculated from thermodynamic integration. The “reference value” corresponds to the free energy difference computed as the area under the blue line in (b), which is a linear fit of the five blue dots. The resulting energy difference between the free energy perturbation and the thermodynamic integration approach is only 0.5 meV/atom.

The key of the proposed approach is the highly optimized MTP that provides a strong overlap with the phase space distribution of the ab initio liquid (see Fig. 1e). This overlap enables more efficient free energy perturbation calculations and avoids the computationally expensive thermodynamic integration calculations based on ab initio MD. To achieve DFT accuracy, the energy difference between the MTP and the ab initio liquid can be computed using Eq. (6), leveraging a few uncorrelated snapshots from the MD trajectories of the MTP. The number of required snapshots depends on the root-mean-square error (RMSE) of the MTP, following the relation (2RMSE/*c*)^2^, where ± *c* represents the target accuracy. This relationship has been tested and discussed in detail in ref. ^19^. According to the RMSE of the MTP (here 2.6 meV/atom for GGA-PBE calculations), to attain an accuracy of 1 meV/atom, only approximately 28 uncorrelated snapshots are needed, as depicted in Fig. 2a.

We compare the computational effort of the proposed approach to the original and hybrid TOR-TILD method (cf. Section “General overview of the methodology”). To have a consistent comparison, the first potential in all three approaches is fitted to solid DFT energies with low-converged DFT parameters; the second potential in both TOR-TILD methods is fitted to liquid DFT energies with low-converged DFT parameters while high-converged DFT parameters are taken for the second potential in the new approach. The required CPU core hours for the three approaches in fitting the potentials and computing a liquid free energy surface on a 5 × 6 (*V*, *T*) grid are listed in Table 1, broken down according to the steps described in Section “Details of the methodology”. Compared to the original TOR-TILD method, the proposed approach is about 20 times faster in the total CPU time. This substantial boost in efficiency stems from the reduction in CPU core hours in Step 4 on computing the black trajectories in Fig. 1e, from 5,526,720 to only 23 CPU core hours, even though the computing efficiency experiences a slight slowdown in Step 2 (Fig. 1c) and Step 3 (Fig. 1d) where MTP calculations are involved. The overall acceleration is attributed to substituting EAM with MTP and TI with FEP. When comparing the new approach with the hybrid TOR-TILD method, the required computational resources are saved by about 80% in calculating a free energy surface on a 5 × 6 (*V*, *T*) grid. Note that the saving factor increases with the number of (*V*, *T*) sampling points. Especially for accurately determining the heat capacity, a denser mesh of the free energy surface is generally required, as the heat capacity is related to the second derivative of the Gibbs energy with respect to temperature. The computational efficiency of the three approaches is also illustrated in (Fig. 1g).

**Table 1:** CPU time in core hours needed by the original and hybrid TOR-TILD method and the newly proposed approach for computing the ab initio free energy surface of liquid TaCrVW on a 5 × 6 (*V*, *T*) grid using LDA

		Original	Hybrid	New
potential fitting	*t*(solid EAM)	15,360	15,360	15,360
	*t*(liquid EAM)	15,360	–	–
	*t*(liquid lowMTP)	–	15,360	15,360
	*t*(liquid highMTP)	–	–	36,000
free energy calculation	*t*(Step 1)	630	630	630
	*t*(Step 2)	25	110	110
	*t*(Step 3)	52	2319	2319
	*t*(Step 4)	5,526,720	990,960	23
	*t*(Step 5)	360,000	360,000	216,000
	*t*(total)	5,918,147	1,384,739	285,802

The time for fitting the potentials is also listed and included in the total time. Steps 1–5 are defined in Section “Details of the methodology”.

The proposed methodology can achieve the same level of ab initio accuracy as the original and hybrid TOR-TILD methods. The energy differences between the MTP and DFT computed by free energy perturbation and thermodynamic integration are relatively small (0.5 meV/atom), as shown in Fig. 2a, b. They result from the chosen convergence accuracy of 1 meV/atom for the thermodynamic sampling in thermodynamic integration and free energy perturbation. These energy differences result in a comparably small melting point shift of 15 K.

### Electronic contribution and its coupling to vibrations

**Fig. 3:**
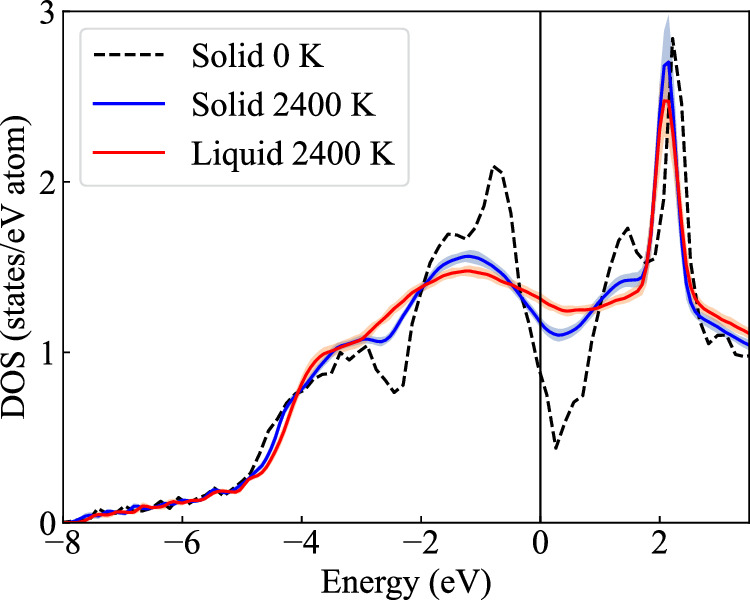
The electronic density of states (DOS) of TaVCrW referenced to the Fermi level. The blue and red lines are, respectively, the DOSs of the solid and liquid at 2400 K and the corresponding equilibrium volume, including the electron-vibration coupling. These DOSs are calculated from a statistically converged set of uncorrelated snapshots. The blue/red shaded areas show the standard deviation from the different snapshots. The black dashed line is for the ideal static lattice at 0 K with an electronic temperature of 2400 K. All DOSs shown here are computed using GGA-PBE.

To analyze the effect of electronic excitations and their coupling to vibrations on the melting properties of TaVCrW, we focus on the DOS of the solid and liquid at 2400 K at the respective equilibrium volume. The GGA-PBE results are plotted in Fig. 3. The DOS of the ideal static lattice at 0 K, with a consistent electronic temperature of 2400 K, is also provided as a reference (depicted by the black dashed line in Fig. 3). This representation highlights how thermal vibrations smooth out the DOS of both the solid and liquid phase at elevated temperatures.

The electronic contribution to the free energy of solid and liquid differs. This disparity results from a gap of 0.16 states/eV ⋅ atom in the DOS of solid and liquid phases at the Fermi level. Correspondingly, this gap translates to an electronic Gibbs energy difference of 4.2 meV/atom between the solid and liquid, leading to a shift in the melting point by 48 K compared to calculations that do not account for the electronic contribution. For the LDA calculations, the electronic Gibbs energy difference between solid and liquid is about 5.8 meV/atom resulting in a melting point shift by 57 K.

### Melting properties

Unlike unary materials, alloys typically exhibit a distinct solidus and liquidus temperature, resulting in a melting interval with upper (liquidus) and lower (solidus) limits, where both solid and liquid phases coexist. Here, the melting point we investigate is the crossing point of the solid and liquid Gibbs energy, called *T*_0_ within the CALPHAD community.

As there are no experimental melting properties for TaVCrW available for a comparison with our DFT results, we extrapolate the melting properties of TaVCrW using the CALPHAD method with Thermo-Calc 2023a^27^. The recent high entropy alloy TCHEA4 database^28^ is utilized which, however, only includes binary descriptions for the subsystems in the Ta-V-Cr-W quaternary system. This is understandable given the lack of adequate experimental ternary phase diagram information. While no general rules exist for assessing the uncertainty of a CALPHAD calculation based on binary system extrapolation, the successful applications of the TCHEA database and its predecessor, TCNI, in designing refractory high entropy alloys^29–34^ indicate the reliability of the database.

**Fig. 4:**
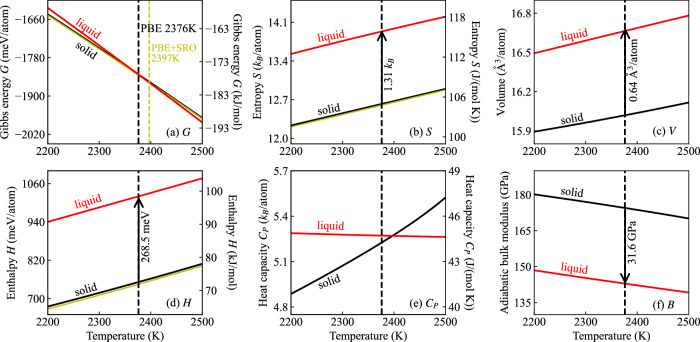
Temperature dependence of thermodynamic properties of solid and liquid TaVCrW from GGA-PBE calculations. **a** Gibbs energy (*G*), (**b**) entropy (*S*), (**c**) volume (*V*), (**d**) enthalpy (*H*), (**e**) heat capacity (*C*_*P*_), and (**f**) bulk modulus (*B*). The yellow lines in (**a**), (**b**), and (**d**) represent the solid Gibbs energy, entropy, and enthalpy, including the SRO contribution, which slightly stabilizes the solid phase and results in a higher melting point of 2397 K.

**Fig. 5:**
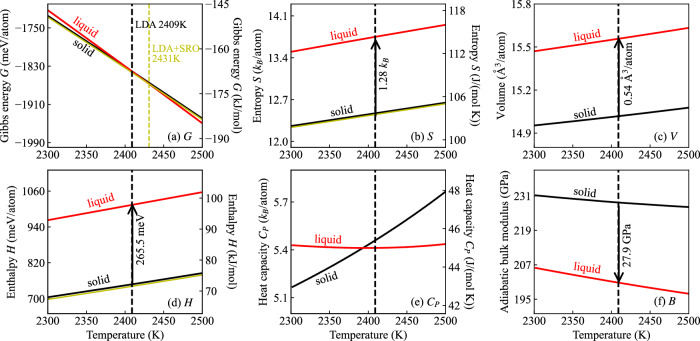
Temperature dependence of thermodynamic properties of solid and liquid TaVCrW from LDA calculations. **a** Gibbs energy (*G*), (**b**) entropy (*S*), (**c**) volume (*V*), (**d**) enthalpy (*H*), (**e**) heat capacity (*C*_P_), and (**f**) bulk modulus (*B*). The yellow lines in (**a**), (**b**), and (**d**) represent the solid Gibbs energy, entropy, and enthalpy, including the SRO contribution, which slightly stabilizes the solid phase and results in a higher melting point of 2431 K.

Figures 4 and 5 depict the temperature dependence of the Gibbs energy (*G*), entropy (*S*), volume (*V*), enthalpy (*H*), heat capacity (*C*_*P*_), and bulk modulus (*B*) for solid (black line) and liquid (red line) TaVCrW from GGA-PBE and LDA. The Gibbs energies are referenced to the internal energy of solid TaVCrW at *T* = 0 K. Table 2 compiles the resulting melting properties for GGA-PBE and LDA in comparison to the CALPHAD method. Our DFT predicted melting points from GGA-PBE and LDA are respectively 2376 K and 2409 K. Both values are lower than the CALPHAD value of 2569 K, but still within the melting interval between 2335 K (solidus) and 2805 K (liquidus) extracted from the CALPHAD method. Our predicted enthalpy of fusion, the entropy of fusion, the heat capacity of both solid and liquid, and the volume change at the corresponding melting point are slightly larger than the calphad values, whereas the predicted volumes for solid and liquid are smaller than the calphad values. Note that the temperature dependence of the bulk modulus for both solid and liquid is also easily accessible with our approach, as shown in Figs. 4f, 5f, where the solid bulk modulus is higher than the liquid one at the corresponding melting point by 31.6 GPa from GGA-PBE and 27.9 GPa from LDA. The existing database within Thermo-Calc does not contain data for bulk modulus for comparison.

**Table 2:** Melting properties of TaVCrW calculated with GGA-PBE and LDA compared to those extracted from the CALPHAD method

	GGA-PBE	LDA	CALPHAD
*T*^m^ (K)	2376	2409	2569
Δ*H*^m^ (kJ/mol)	25.9	25.6	23.8
Δ*H*^m^ (meV/atom)	268.5	265.5	246.3
Δ*S*^m^ (J/(mol K))	10.9	10.63	9.25
Δ*S*^m^ (*k*_B_/atom)	1.31	1.28	1.11
${C}_{P,{\rm{solid}}}^{{\rm{m}}}$ (J/(mol K))	43.45	45.39	42.58
${C}_{P,{\rm{liquid}}}^{{\rm{m}}}$ (J/(mol K))	43.84	44.99	43.55
Δ*V*^m^ (Å^3^/atom)	0.64	0.54	0.51
${V}_{{\rm{solid}}}^{{\rm{m}}}$ (Å^3^/atom)	16.02	15.02	16.37
${V}_{{\rm{liquid}}}^{{\rm{m}}}$ (Å^3^/atom)	16.66	15.56	16.88

Notably, even though the absolute values of the thermodynamic properties for both the solid and liquid are different between GGA-PBE and LDA, the temperature dependence is similar, as shown in Figs. 4, 5, which is consistent with our previous finding for tungsten^10^.

### Configurational entropy and impact of short-range order

We assume ideal mixing of the elements for the solid and liquid free energy calculations. To quantify the degree of short-range order and its possible impact, we performed simulations employing on-lattice machine learning potentials, namely the low-rank potentials, see Section “Short-range order calculations” for the technical details. We focus on the high-temperature regime of 2200–2500 K as in Fig. 4, relevant for melting properties. To allow for efficient computation, we have chosen a temperature of 2260 K, which is about 100 K below the GGA-PBE computed melting temperature, to fix the volume and electronic free energy contributions.

We computed the configurational part of the internal energy, ${{\Delta }}{U}_{{\rm{conf}}}^{{\rm{solid}}}$, entropy, ${{\Delta }}{S}_{{\rm{conf}}}^{{\rm{solid}}}$, and free energy, ${{\Delta }}{F}_{{\rm{conf}}}^{{\rm{solid}}}$ referenced to the ideal random alloy. These quantities allow for a direct evaluation of the impact of SRO as compared to the ideal random alloy assumption. The internal energy is directly accessible via the Monte Carlo simulations. The entropy contribution is computed by integrating the heat capacity from high temperatures downwards^35^.

Due to SRO, the configurational entropy is slightly smaller than the ideal configurational entropy of $\ln (4)$. It, therefore, results via −*TS* in a positive (destabilizing) entropy-driven energy contribution of around 5–6 meV/atom. However, SRO also decreases the internal energy by about 8–9 meV/atom (Fig. 4d). Hence the resulting free energy of the solid is overall stabilized by about 2.4 meV/atom, Fig. 4a, resulting in a slight shift of the melting temperature as discussed further below.

## Discussion

A hierarchical approach for efficient calculations of the liquid free energy of high entropy alloys with ab initio accuracy has been introduced. The approach integrates EAM, MTP, and DFT to achieve progressively increasing accuracy while maintaining maximally optimized computational speed. This combination improves the overall computational efficiency by saving 80% of the computational resources as compared to similarly accurate methods while retaining accuracy in the sub-meV range. The primary innovation lies in leveraging the advantages of MTP and replacing the expensive thermodynamic integration with free energy perturbation calculations. The substantial reduction in computing effort makes the approach attractive for high-throughput calculations of melting properties of multi-component alloys with ab initio accuracy.

The approach has been applied to the bcc refractory high entropy alloy TaVCrW, for which currently no experimental data of melting properties exist. The results obtained with the new method align closely with those from the TOR-TILD methodology (deviations < 0.5 meV/atom). To put the predicted properties into perspective, we have compared the data with those computed with the CALPHAD method using Thermo-Calc 2023a. Both our DFT predictions are below the CALPHAD extracted value (2569 K) by 193 K for GGA-PBE and 160 K for LDA. This discrepancy is not a direct shortcoming of the proposed methodology since the established TOR-TILD method, which does not rely on free energy perturbation and can be considered as a higher-level reference, gives the same results. To further support this statement, we have performed an additional benchmark for the VW system. The VW system is simpler than the present TaVCrW alloy, the available calphad data are very reliable, and its melting properties have been intensively assessed previously^10^. The benchmark for VW leads to the same conclusion, i.e., that the here proposed method does not introduce any additional uncertainty into the results. Hence, the observed discrepancy between calphad and DFT must be of a different nature. A few uncertainties that may contribute to this discrepancy are listed in the following.

First, for the CALPHAD extracted melting point, the input data in the high entropy alloy database for parametrization includes only sub-binary alloy information for TaVCrW. Although direct evidence for the uncertainty of the CALPHAD predicted melting point of the TaVCrW alloy is lacking, we can reasonably speculate that the error is within ± 100 K, based on comparing calphad predicted values with those from experimental and theoretical data for similar refractory alloy systems^34,36,37^.

Second, for the DFT calculations, the estimated error in the computed Gibbs energy arising from statistics and fitting is about 2.5 meV/atom for both the solid and liquid. For the free energy approach, an error of 1 meV/atom in either solid or liquid phase would introduce a ~10 K shift in the melting point prediction. We, therefore, estimate the maximum numerical error in the DFT predicted melting points as ±50 K.

Third, high-temperature magnetic fluctuations have not been included. Cr and V, for example, are treated as non-magnetic elements at the investigated temperature range. We have performed additional spin-polarized test calculations using GGA-PBE based on high-temperature MD snapshots, revealing a negligible impact. However, it was reported that standard DFT employing GGA-PBE or LDA fails to account for strong magnetic fluctuations in bcc Cr^38^. More elaborate treatment of magnetism may resolve this issue, which is, however, beyond the scope of the present work.

Fourth, the SRO contribution to the solid Gibbs energy stabilizes the solid phase by 2–3 meV/atom at *T*_m_. This contribution is relatively small because the melting temperature is nearly twice the chemical order-disorder temperature^39^. The effect on the calculated melting temperature is approximately 25 K. While SRO contributions may have a more significant impact in alloys with strong ordering and greater SRO near the melting temperature, SRO does not significantly affect the computation of the melting temperature in the current alloy.

Overall, considering the discussed uncertainty of the CALPHAD extrapolated value and the accumulated possible uncertainties from the DFT predictions, we achieve a reasonable agreement for the melting temperature value of bcc TaVCrW.

Apart from the quantitative comparison, a crucial merit of the approach is that it provides physical insights into how different thermal contributions affect the melting properties of materials, in particular, the electronic excitations and their coupling to lattice vibrations. In our previous work^10^, we observed a significant impact of electronic excitations in tungsten resulting in a large melting point shift (178 K for GGA-PBE and 158 K for LDA). In the present work, given that tungsten constitutes 1/4 of the investigated TaVCrW alloy, the induced change in melting point is smaller but still significant (48 K for GGA-PBE and 57 K for LDA) and again highlights the necessity to include the electronic contribution into melting point considerations.

**Fig. 6:**
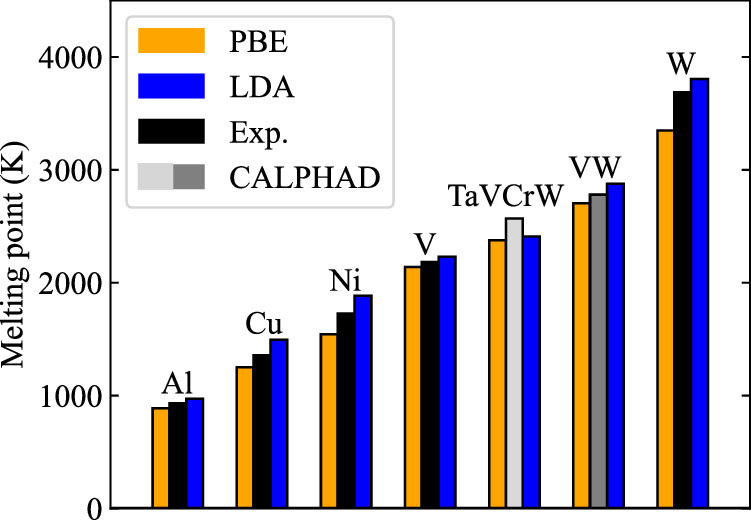
Performance of the standard exchange-correlation functionals GGA-PBE and LDA in predicting the melting point. The DFT data for Cu are from ref. ^16^, for Al and Ni from ref. ^17^, for V, VW, and W from ref. ^10^. The black bars indicate the experimental melting points. The gray and light gray bars are the melting points extrapolated from the CALPHAD method (crossing points of the liquid and solid Gibbs energy corresponding to *T*_0_ used in the CALPHAD community). Explicit experimental melting data is available for binary VW (gray bar). In contrast, for TaVCrW the extrapolated melting point from the calphad method (light gray bar) is an approximate theoretical prediction based on available binary alloys in the database.

The efficiency of the approach allows us also to evaluate the performance of different exchange-correlation functionals on melting property predictions within the DFT framework, which would be otherwise a computationally much more challenging task. Our previous works^10,16,17^ have shown that GGA-PBE and LDA provide an ab initio confidence interval for the experimental melting points, i.e., GGA-PBE predicts a lower melting boundary and LDA a higher melting boundary, as shown in Fig. 6. The reason is attributed to the underbinding / overbinding property of GGA-PBE / LDA. For TaVCrW, using the CALPHAD value as a proxy for the experiment, this empirical rule seems to not hold anymore. However, this does not necessarily invalidate this rule but could be related to the aforementioned uncertainties in the CALPHAD value. To elucidate this point further, we considered the performance of the exchange-correlation functionals for the equilibrium lattice constants at *T* = 0 K. Already for the pure elements, the underbinding / overbinding tendency of GGA-PBE / LDA, which generally predicts larger / smaller equilibrium lattice constants compared to experimental values for most unaries, does not hold for unary V^10,40,41^ and Cr^41,42^ anymore, where both GGA-PBE and LDA predict smaller equilibrium lattice constants at *T* = 0 K. This discrepancy may propagate into the here-considered TaVCrW multi-component alloy. It would be therefore instructive to first elaborate on different methods (e.g., dynamical mean-field theory), capable of resolving the *T* = 0 K discrepancies for the unaries^10,43,44^, before employing these computationally more expensive approaches for melting point calculations. We also note that other DFT treatments can be integrated into the proposed free energy perturbation approach since it requires only the computation of energies but not forces.

The proposed method offers a computationally efficient (80% of resource savings) and highly accurate (within the sub-meV range) way to determine the melting properties of multi-component alloys. The method is thus an alternative to challenging and not always feasible experiments as well as to rough estimates based on linear averages of unary properties. Moreover, it can be also used as an accurate reference for designing machine-learning potentials, which could provide a more approximate but faster alternative for screening the compositional space. In any case, being able to explore melting properties for a wide range of compositions in chemically complex alloys provides valuable opportunities for materials design.

## Methods

### Details of the methodology

We sketch the entire workflow of the proposed method in five steps and compare the steps to those of the original TOR-TILD method.

**Step 1:** The first step remains the same as in the original TOR-TILD method. We apply the interface method^45^, also called coexistence approach, to compute the melting temperature of the EAM, ${T}_{{\rm{EAM}}}^{{\rm{m}}}$. The employed interface structure is shown in Fig. 1a. The efficiency of EAM allows us to perform calculations on sufficiently large supercells and long timescales. Here, a 16 × 16 × 32 supercell (16,384 atoms) is used for TaVCrW with the simulation time up to 50 ps with a timestep of 1 fs. The simulations are carried out with our previously developed automated pyiron workflow^5^. Determining the melting point of an empirical potential with high precision requires performing a few tens of calculations with different initial random seeds. The resulting statistical distribution of melting points closely resembles a Gaussian function, and the mean is used as a precise prediction, as shown in Fig. 1a. The corresponding standard error is well below 1 K and can be neglected in the final DFT melting point prediction.

At ${T}_{{\rm{EAM}}}^{{\rm{m}}}$ the volume of solid, ${V}_{{\rm{EAM}}}^{{\rm{m,solid}}}$, and the volume of liquid, ${V}_{{\rm{EAM}}}^{{\rm{m,liquid}}}$, are respectively computed by *NPT* MD simulations using the EAM potential. The solid free energy ${F}_{{\rm{EAM}}}^{{\rm{solid}}}$(${V}_{{\rm{EAM}}}^{{\rm{m,solid}}},{T}_{{\rm{EAM}}}^{{\rm{m}}}$) is then calculated by thermodynamic integration from an effective quasiharmonic (QH) reference (computed in ref. ^18^) through

2 $$\begin{array}{ll}{F}_{{\rm{EAM}}}^{{\rm{solid}}}\left({V}_{{\rm{EAM}}}^{{\rm{m,solid}}},{T}_{{\rm{EAM}}}^{{\rm{m}}}\right)\,=\,{F}_{{\rm{QH}}}^{{\rm{solid}}}({V}_{{\rm{EAM}}}^{{\rm{m,solid}}},{T}_{{\rm{EAM}}}^{{\rm{m}}})\\\qquad\qquad\qquad\qquad\qquad\,\,+\displaystyle\mathop{\int}\nolimits_{0}^{1}d\lambda {\left\langle {E}_{{\rm{EAM}}}^{{\rm{solid}}}-{E}_{{\rm{QH}}}^{{\rm{solid}}}\right\rangle}_{\lambda ,{T}_{{\rm{EAM}}}^{{\rm{m}}}},\end{array}$$

as shown in Fig. 1b. Here, 〈…〉_*λ*,*T*_ denotes the thermodynamic average at a temperature *T* and coupling *λ*. A simple Einstein model has been also tested as a reference. The difference in free energy between an effective QH and an Einstein reference is only 0.7 meV/atom, indicating that using different references at this stage has a negligible impact on computational accuracy. Once ${F}_{{\rm{EAM}}}^{{\rm{solid}}}({V}_{{\rm{EAM}}}^{{\rm{m,solid}}},{T}_{{\rm{EAM}}}^{{\rm{m}}})$ is obtained, ${F}_{{\rm{EAM}}}^{{\rm{liquid}}}$(${V}_{{\rm{EAM}}}^{{\rm{m,liquid}}},{T}_{{\rm{EAM}}}^{{\rm{m}}}$) is simultaneously available based on the relation *G*(*P*, *V*) = *F*(*V*, *T*) + *PV* and the fact that the liquid Gibbs energy equals to the solid one at the melting point and constant pressure (here *P* = 0 GPa). Note that the melting properties at different pressures can be easily accessed by adding the *PV* term to the Helmholtz energies.

**Step 2:** In this step using ${F}_{{\rm{EAM}}}^{{\rm{liquid}}}$(${V}_{{\rm{EAM}}}^{{\rm{m,liquid}}},{T}_{{\rm{EAM}}}^{{\rm{m}}}$) from Step 1 as a starting point, the liquid free energy of the MTP is obtained by performing thermodynamic integration through

3 $$\begin{array}{ll}{F}_{{\rm{MTP}}}^{{\rm{liquid}}}\left({V}_{{\rm{EAM}}}^{{\rm{m,liquid}}},{T}_{{\rm{EAM}}}^{{\rm{m}}}\right)\,=\,{F}_{{\rm{EAM}}}^{{\rm{liquid}}}\left({V}_{{\rm{EAM}}}^{{\rm{m,liquid}}},{T}_{{\rm{EAM}}}^{{\rm{m}}}\right)\\\qquad\qquad\qquad\qquad\qquad\quad+\displaystyle\mathop{\int}\nolimits_{0}^{1}d\lambda {\left\langle {E}_{{\rm{MTP}}}^{{\rm{liquid}}}-{E}_{{\rm{EAM}}}^{{\rm{liquid}}}\right\rangle }_{\lambda ,{T}_{{\rm{EAM}}}^{{\rm{m}}}},\end{array}$$

as demonstrated in Fig. 1c. MTP is involved in this step compared to the original TOR-TILD using two EAMs.

**Step 3:** In this step, using ${F}_{{\rm{MTP}}}^{{\rm{liquid}}}$ (${V}_{{\rm{EAM}}}^{{\rm{m,liquid}}},{T}_{{\rm{EAM}}}^{{\rm{m}}}$) from Step 2 as a starting point, we unfold the liquid free energy surface of the MTP by integrating the pressure *P*(*V*, *T*) along the volume dimension using

4 $$\begin{array}{ll}{F}_{{\rm{MTP}}}^{{\rm{liquid}}}\left(V,{T}_{{\rm{EAM}}}^{{\rm{m}}}\right)\,=\,{F}_{{\rm{MTP}}}^{{\rm{liquid}}}\left({V}_{{\rm{EAM}}}^{{\rm{m}}},{T}_{{\rm{EAM}}}^{{\rm{m}}}\right)\\\qquad\qquad\qquad\qquad+\displaystyle\mathop{\int}\nolimits_{{V}_{{\rm{EAM}}}^{{\rm{m}}}}^{V}P\left({V}^{{\prime} },{T}_{{\rm{EAM}}}^{{\rm{m}}}\right)\,d{V}^{{\prime} },\end{array}$$

and integrating the internal energy *U*(*V*, *T*) along the temperature dimension using

5 $$\begin{array}{ll}\frac{{F}_{{\rm{MTP}}}^{{\rm{liquid}}}(V,T)}{{k}_{{\rm{B}}}T}\,=\,\frac{{F}_{{\rm{MTP}}}^{{\rm{liquid}}}\left(V,{T}_{{\rm{EAM}}}^{{\rm{m}}}\right)}{{k}_{{\rm{B}}}{T}_{{\rm{EAM}}}^{{\rm{m}}}}\\\qquad\qquad\quad+\displaystyle\mathop{\int}\nolimits_{{T}_{{\rm{EAM}}}^{{\rm{m}}}}^{T}d\left(\frac{1}{{T}^{{\prime} }}\right)U(V,{T}^{{\prime} }),\end{array}$$

where *k*_B_ is the Boltzmann constant. This step is illustrated in Fig. 1d. Here, the MTP fully substitutes EAM compared to the original TOR-TILD method. As applying MTP is much slower than using EAM, a relatively small but still converged supercell size of 12 × 12 × 12 (3456 atoms) is employed instead of a supercell size of 16 × 16 × 16 (8192 atoms) when using EAM.

**Step 4:** In the original (and hybrid) TOR-TILD method, this step requires ab initio MD simulations for the thermodynamic integration calculations at various *λ* values, volumes, and temperatures. In the present method, only MTP MD simulations are carried out to generate the trajectories at a set of volumes and temperatures (the black line in Fig. 1e). The applied supercell size is 4 × 4 × 4 (128 atoms) and the same as used for the DFT calculations in Step 5. Here, the computational effort is tremendously reduced by a magnitude of 10^4^ in CPU core hours compared to the hybrid TOR-TILD, see Table 1.

**Step 5:** In this last step, ab initio accuracy is achieved. We use the set of uncorrelated snapshots from the MTP MD trajectories generated in Step 4 to calculate the change in free energy from MTP to DFT with high converged parameters via perturbation theory. The respective DFT energies are represented by the red dots in Fig. 1e. The free energy difference between the MTP and DFT can be calculated using

6 $${\Delta F}_{{\rm{MTP}}\to{\rm{DFT}}}^{{\rm{liquid}}}\,=-{k}_{{\rm{B}}}T{\rm{ln }}{\left\langle {e}^{-\frac{1}{{k}_{{\rm{B}}}T}({E}^{{\rm{DFT}}}-{E}^{{\rm{MTP}}})}\right\rangle }_{\rm{MTP}},$$

where *E*^DFT^ and *E*^MTP^ are the energies of the snapshots obtained with DFT and MTP, respectively. Importantly, the variance of the difference in these energies is very small, and the free energy in Eq. (6) converges quickly. This highlights the high quality of the MTP for performing free energy perturbation calculations. The electronic contribution and the impact of atomic vibrations on it are included by utilizing finite temperature DFT for computing *E*^DFT^, i.e., by setting the electronic temperature equal to the MD temperature. The resulting change in the energy only introduces a constant shift but neither affects the variance nor the convergence of the free energy.

In the original (and hybrid) TOR-TILD method, free energy perturbation theory is also applied, in the sense of the up-sampling technique^21^, in order to correct the free energy computed with low-converged DFT parameters to high-converged parameters. Note that this step in the original (and hybrid) TOR-TILD method is done *in addition* to the thermodynamic integration involving the DFT-based MD.

### Potential fitting: EAM and MTP

We utilized the MEAMfit code^46^ for fitting the EAM potential. The EAM was fitted only to the solid DFT energies from ab initio MD trajectories computed with low-converged DFT parameters (cutoff energy of 300 eV and *k*-mesh of 2 × 2 × 2). Four volumes from the relevant volume range were selected for generating the training data at 2500 K. This potential was used in Steps 1 and 2. The interface MD calculations, including the liquid, are stable, even though the EAM was fitted only to the solid phase. An accurate (with respect to DFT) prediction of the EAM melting point is not required due to the following corrections to MTP and DFT.

For fitting the MTP, we employed the MLIP package^47^ implemented in pyiron^48^ within the potential fitting module^49^. We employed a two-stage fitting procedure to optimize efficiency. First, we fitted a low-quality MTP with level 20 (lowMTP) to liquid ab initio MD trajectories computed with low-converged DFT parameters, the same as used for fitting the EAM. The fitting temperature was 2500 K, and five volumes from the relevant volume range were selected. The lowMTP was then used to generate new MD trajectories at the same temperature and volumes, followed by static DFT calculations with high-converged DFT parameters (cutoff energy of 450 eV and *k*-mesh of 4 × 4 × 4) for a set of uncorrelated MTP MD snapshots. Second, we fitted a high-quality MTP with level 24 (highMTP) using the DFT energies from the static DFT calculations in the preceding step. The RMSE of the highMTP for the GGA-PBE liquid calculations is 2.6 meV/atom, which is similar to 2.4 meV/atom obtained previously for solid TaCrVW^18^. The highMTP was used in Steps 2, 3, and 4.

### Short-range order calculations

To analyze the short-range order in the solid, we utilized low-rank interatomic potentials (LRP)^50,51^ as an interaction model in canonical Monte Carlo (MC) simulations. The LRPs belong to a class of on-lattice machine-learning potentials and have been proven to perform very efficiently for computing SRO parameters in multi-component alloys^35,52–55^. We evaluated different ranks and have chosen a rank of 3 for the final evaluation. The training was performed on 128-atom cells by training a set of ten independent potentials to evaluate the uncertainty. During the iterative retraining process, 324 and 36 configurations were added to the final training and validation sets, respectively. The training and validation errors are below 1 meV/atom. The Monte Carlo simulations were carried out with periodic boundary conditions. We mainly focused on the 2000–2400 K temperature range where the alloy remains disordered. The simulations were carried out for systems containing 128 and 5488 atoms, i.e., 4 × 4 × 4 and 14 × 14 × 14 lattice units, based on a two-atom primitive BCC cell. The number of MC steps (atom-atom swap attempts) was 20,000 times the number of atoms in the corresponding supercell. The burn-in approach^56^ was utilized for thermal equilibration, i.e., for each temperature, the first half of MC steps was neglected. Test calculations with 50,000 MC steps per atom were performed to corroborate the findings. From the MC simulations, the internal energy and specific heat capacity are directly accessible. To compute the configurational contribution to the entropy, we followed the approach of ref. ^35^, i.e., we integrated the specific heat capacity and subtracted it from a high-temperature reference state taken at 10,000 K representing the ideal disordered state with entropy $\ln 4{k}_{B}$.

The DFT calculations for the training were performed at a lattice constant of 3.171 Å and using an electronic smearing parameter of 0.195 eV, which corresponds to the (GGA-PBE) values at 2260 K, about 100 K below the predicted melting temperature. The details of the other DFT parameters are as in Section “Computational details of the DFT and MD calculations”. For computational efficiency, the plane wave cutoff and *k* point mesh were reduced to 270 eV and 3 × 3 × 3, respectively. Test calculations from the training set revealed that more accurate parameters resulted in a constant energy shift with energy variation among the configurations below 0.2 meV/atom.

### Computational details of the DFT and MD calculations

For the DFT calculations, we used the projector-augmented wave (PAW) method^57^ as implemented in the VASP software package^58–61^. PAW potentials for Ta, Cr, V, and W treating *p* electrons as the valence states were used. LDA and GGA were employed for the exchange-correlation functional, with the Perdew-Burke-Ernzerhof (PBE)^62^ parametrization for GGA. The data of solid free energy from GGA-PBE were taken from ref. ^18^. The solid free energy for LDA was calculated with direct upsampling in the present work, as well as the liquid free energy for both GGA-PBE and LDA using the new approach. The sets of explicitly DFT computed volume and temperature points for both solid and liquid in the present work are given in Table 3. The DFT calculations were performed in a 4 × 4 × 4 supercell with 128 atoms. For the solid, a 128-atom SQS structure was used. For liquid, the SQS structure was heated up to a high temperature, e.g., 3000 K, to trigger the liquid phase. The explicitly computed DFT points were used as input to fit polynomials up to third order to obtain an analytical description of the free energy surface as a function of volume and temperature. The plane wave cutoff and *k* point mesh (Monkhorst-Pack^63^) were set to 450 eV and 4 × 4 × 4, respectively. For details on the fitting procedure of the corresponding GGA-PBE solid free energy surface and the respective convergence parameters, we refer to ref. ^18^.

**Table 3:** Mesh of explicitly computed volumes, *V* (per atom), and temperatures, *T*, that are used for obtaining the free energy surface

Solid		
LDA	*V* (Å^3^)	13.9, 14.19, 14.47, 14.75, 15.04
	*a* (Å)	3.03, 3.05, 3.07, 3.09, 3.11
	*T*(K)	2300, 2400, 2500, 2600, 2700, 2800

Liquid		
GGA-PBE	*V* (Å^3^)	15.04, 15.33, 15.63, 15.93, 16.23
	*a* (Å)	3.11, 3.13, 3.15, 3.17, 3.19
	*T*(K)	2200, 2400, 2600, 2800
	*V* (Å^3^)	14.47, 14.75, 15.04, 15.33, 15.63
LDA	*a* (Å)	3.07, 3.09, 3.11, 3.13, 3.15
	*T*(K)	2300, 2400, 2500, 2600, 2700, 2800

The volumes are additionally expressed in terms of a corresponding bcc lattice constant, *a* = (2*V*)^1/3^.

For the reference potential calculations, we used the LAMMPS software package^64^. For fitting the liquid free energy surface of our potential, we used the same volumes as for the DFT calculations (see Table 3), but at a denser temperature mesh (steps of 20 K). For the MD simulations using the specially designed MTP we used a time step of 5 fs and the Langevin thermostat with a friction parameter of 0.01 fs^−1^ to control the temperature.

## Data Availability

The authors declare that the data supporting the findings of this study are available within the article and its supplementary information files or from the corresponding authors on reasonable request.
